# Identifying radiation-induced survivorship syndromes affecting bowel health in a cohort of gynecological cancer survivors

**DOI:** 10.1371/journal.pone.0171461

**Published:** 2017-02-03

**Authors:** Gunnar Steineck, Viktor Skokic, Fei Sjöberg, Cecilia Bull, Eleftheria Alevronta, Gail Dunberger, Karin Bergmark, Ulrica Wilderäng, Jung Hun Oh, Joseph O. Deasy, Rebecka Jörnsten

**Affiliations:** 1 Division of Clinical Cancer Epidemiology, Department of Oncology, Institute of Clinical Sciences, Sahlgrenska Academy at the University of Gothenburg, Gothenburg, Sweden; 2 Department of Oncology and Pathology, Division of Clinical Cancer Epidemiology, Karolinska Institutet, Stockholm, Sweden; 3 Institute of caring Sciences, Ersta Sköndal University College, Stockholm, Sweden; 4 Department of Medical Physics, Memorial Sloan Kettering Cancer Center, New York, United States of America; 5 Chalmers University of Technology, Gothenburg, Sweden; University Hospital Llandough, UNITED KINGDOM

## Abstract

**Background:**

During radiotherapy unwanted radiation to normal tissue surrounding the tumor triggers survivorship diseases; we lack a nosology for radiation-induced survivorship diseases that decrease bowel health and we do not know which symptoms are related to which diseases.

**Methods:**

Gynecological-cancer survivors were followed-up two to 15 years after having undergone radiotherapy; they reported in a postal questionnaire the frequency of 28 different symptoms related to bowel health. Population-based controls gave the same information. With a modified factor analysis, we determined the optimal number of factors, factor loadings for each symptom, factor-specific factor-loading cutoffs and factor scores.

**Results:**

Altogether data from 623 survivors and 344 population-based controls were analyzed. Six factors best explain the correlation structure of the symptoms; for five of these a statistically significant difference (P< 0.001, Mann-Whitney U test) was found between survivors and controls concerning factor score quantiles. Taken together these five factors explain 42 percent of the variance of the symptoms. We interpreted these five factors as radiation-induced syndromes that may reflect distinct survivorship diseases. We obtained the following frequencies, defined as survivors having a factor loading above the 95 percent percentile of the controls, *urgency syndrome* (190 of 623, 30 percent), *leakage syndrome* (164 of 623, 26 percent), *excessive gas discharge* (93 of 623, 15 percent), *excessive mucus discharge* (102 of 623, 16 percent) and *blood discharge* (63 of 623, 10 percent).

**Conclusion:**

Late effects of radiotherapy include five syndromes affecting bowel health; studying them and identifying the underlying survivorship diseases, instead of the approximately 30 long-term symptoms they produce, will simplify the search for prevention, alleviation and elimination.

## Introduction

When cancer treatment has eliminated the last malignant cell, the long-term unwanted consequences of the treatment, sadly enough, become lifelong threats to the cured cancer survivor’s health [1]. Concerning cancer situated in the thorax, the threats are handled primarily as a survivorship disease such as, for example, radiation-induced heart failure or cardiac infarction. Such an approach has advantages as compared to using single symptoms as an end point in clinical research [2]. Concerning cancer situated in the pelvic cavity a large number of symptoms have been documented as radiation-induced late effects [3]; it is unclear, however, if these symptoms should be seen as manifestations of one or several survivorship diseases [4–6]. We have no nosology for radiation-induced survivorship diseases and we do not know which disease decreasing bowel health produces which symptoms. Identifying radiation-induced syndromes among cancer survivors may give insight into the presence of distinct radiation-induced survivorship diseases and the symptoms they produce.

Modern cancer treatment clearly differs from that given 50 years ago [7]. Surgical mortality has declined, e.g. due to anesthesiological achievements and refined surgical techniques. We now have access to an ever increasing number of cytotoxic drugs and biological response modifiers [8]. New technology for imaging of the patient and tumor and for administration of ionizing radiation has made it possible to dramatically increase the ratio between the dose of ionizing radiation to the tumor and unwanted doses to surrounding normal tissue during radiotherapy, an improvement often making it possible to increase doses to the tumor [9]. All these success stories have produced a historically new situation with a large growth in the number of aging cancer survivors—probably in the vicinity of 21 million in Europe—as well as an increasing number of survivors with treatment-induced survivorship diseases that decrease long-term bowel health.

Possibly we already have the means to prevent, alleviate or eradicate a large part of the survivorship diseases that decrease bowel health, means including better dose plans, dietary changes, probiotics or drugs. We just lack the knowledge needed to employ these means satisfactorily. In our division we have developed clinimetric methods to document patient-reported long-term symptoms one by one (atomized symptom documentation) [10–13]. These methods give data sets which can be used to disentangle syndromes that may reflect radiation-induced survivorship diseases that produce the symptoms. Moreover, during the 1990s and 2000s by and large all patients with cancer in Sweden in a certain geographical region were treated at the same single clinic for radiotherapy. All residents in Sweden have personal identity numbers which together with population-based registers makes it easy to identify and follow up long-term cancer survivors by using postal questionnaires. Together with a literate and cooperative population we thus have the possibility to retrieve truly population-based information with high accuracy. Benefiting from this situation, we have retrieved patient-reported information on 28 long-term gastrointestinal symptoms among gynecological cancer survivors treated with radiotherapy [14]. Using a novel modification of factor analysis, we here disentangle syndromes among these, syndromes that may be related to distinct radiation-induced survivorship diseases. We also investigate which long-term symptoms to be included in which syndrome.

## Methods

### Survivors and controls

Dunberger and co-workers describe the data collection in detail [14–16]. Between 1991 and 2003 altogether 1800 women were treated with external pelvic radiotherapy for a gynecological malignancy at two clinics in Sweden, by and large corresponding to all relevant patients in two geographical regions. We excluded survivors born 1927 or earlier and who could not understand Swedish (S1 Fig).

### Data collection

#### Qualitative phase

In semistructured interviews with 23 women, we attempted to document all of the symptoms the survivors had at the time. A secretary transcribed the interviews verbatim, and we sorted the information into groups of statements reflecting specific atomized long-term symptoms. Based on this, we constructed a study-specific questionnaire with wording as close as possible to those of the survivors. For example, when asking for flatulence we gave both of the Swedish words corresponding to fart and wind in English. We asked for occurrence and, selectively, intensity and duration of the symptom [13]. For example, in answering “Do you have uncontrolled loud wind (fart)” answering categories were “No”, “Yes, occasionally”, “Yes, at least once a month,” “Yes, at least once a week,” “Yes, at least 3 times a week,” and “Yes, at least once a day” (a person-incidence scale).

#### Data collection

All survivors received a letter and a telephone call before we sent out the questionnaire. Three weeks after posting the questionnaire we sent a thank-you-and-reminder card and, when appropriate, made reminding telephone calls. All actions were taken by neutral third-party secretariat [17]; none of the previously involved health-care professionals were involved or had access to the data.

Ethics statement, as endorsed by the Ethical Review Board, completing the questionnaire and posting it to us was considered as a written consent of participation. The study was approved by the Regional Ethical Review Board (2005/1424-31/4), Stockholm, Sweden.

### Statistical analysis

#### Overview

To make the analysis blinded, the two involved statisticians used variable names such as “V43” with no labels referring to the symptoms during the programming. We used a modified Exploratory Factor Analysis to define the number of factors that best describe the correlation matrix of the data, factor loadings, factor-specific cutoffs for factor loadings and factor scores (for details, please see the statistical appendix S1 File) [18]. Having ordinal data, we consistently used Spearman’s rank correlation coefficient as input to the Exploratory Factor Analysis. Parameters were estimated using maximum likelihood estimation [19].

#### Number of factors

In an effort to avoid over- and underestimation of the optimal number of factors, we used Parallel analysis with 10 000 permutations of the data as well as a version of Kaiser’s rule, based on 10 000 non-parametric bootstrap estimates [20–22]. Both methods are based on an investigation of eigenvalues of the estimated data correlation matrix.

#### Factor-specific factor-loading cutoffs

We formulated a tailor-made method for setting factor-specific cutoffs. We evaluated this method and made decisions on parameter values based on simulated data from nine distributions of known factor structures.

#### Factor-score comparison

We compared quantiles of the factor scores of the survivors and population controls. The factor scores were calculated using only symptoms with factor loadings greater in magnitude than the previously determined factor-specific factor-loading cut-offs. First the two data sets were imputed using mode imputation. The imputed data sets were then combined into one data set that was standardized and the survivor-specific factor scores were calculated as linear combinations of the observations for the survivor and the factor-loadings associated with a specific factor that were larger in magnitude than the factor-specific cutoff. We finally compared, for each factor, the factor-score quantiles of the survivors and the population controls using the Mann-Whitney U test.

## Results

As seen in the flow chart (S1 Fig), 650 (79%) of 823 eligible gynecological-cancer survivors and 344 (72%) of 478 eligible matched population-based controls returned a questionnaire. Of the gynecological-cancer survivors we excluded seven with missing information on more than 30 percent of the 28 variables reflecting long-term symptoms and 20 having a bowel stoma, leaving us with 623 survivors for the present analysis. Table 1 shows that most survivors were in the age 60 to 69 category, 63 percent were married or had a partner and 30 percent had a university education. A coding error made the population controls on average younger than the survivors; the difference in mean age was 6.2 years. Any relation between age and symptom occurrence among the population-based controls (data not shown) is weak if at all present.

**Table 1:** Certain characteristics for 623 gynecological-cancer survivors and 344 population-based controls.

GYNECOLOGICAL CANCER SURVIVORS (N = 623) AND CONTROLS (N = 344)					
	No. (%)			No. (%)	
	Survivors	Controls		Survivors	Controls
Age at follow-up*—years			**Pelvic floor injury**^₸^^,^^£^		
-49	66 (11)	102 (30)	Yes	111 (17)	101 (29)
50–59	100 (16)	80 (23)	Not stated	14 (2)	2 (1)
60–69	245 (40)	78 (23)	**Intercurrent diseases**^£^		
70-	212 (33)	82 (24)	Previous abdominal surgery	253 (41)	156 (45)
Not stated	0 (0)	2 (1)	Not stated	31 (5)	0 (0)
**Marital status**			Diabetes mellitus	54 (9)	17 (5)
Married or living with partner	355 (57)	220 (64)	Not stated	5 (1)	6 (2)
Widow	81 (13)	37 (11)	Hypertension	220 (35)	91 (27)
Has partner but lives alone	36 (6)	22 (6)	Not stated	13 (2)	3 (1)
Single	149 (24)	65 (19)	Heart failure	32 (5)	8(2)
Not stated	2 (0)	0 (0)	Not stated	13 (2)	3 (1)
**Education**			Angina pectoris	32 (5)	11 (3)
Elementary school	194 (31)	69 (20)	Not stated	13 (2)	3 (1)
Secondary school	238 (38)	146 (42)	Cardiac infarction	18 (3)	5 (2)
College or university	190 (30)	127 (37)	Not stated	13 (2)	3 (1)
Not stated	1 (0)	2 (1)	Crohn's disease treatment	1 (0)	0 (0)
**Employment**			Not stated	18 (3)	12 (4)
Student	6 (1)	2 (1)	Ulcerative colitis	4 (1)	7 (2)
Unemployed	14 (2)	6 (2)	Not stated	33 (5)	17 (5)
Employed	204 (33)	188 (55)	IBS^₽^ treatment	23 (4)	13 (4)
Housewife, other	12 (2)	5 (2)	Not stated	20 (3)	12 (4)
On sick leave	11 (2)	10 (3)	Hemorrhoids treatment	57 (9)	45 (13)
Disability pension	55 (9)	15 (4)	Not stated	24 (4)	22 (6)
Retired	318 (51)	117 (34)	Lactose intolerance	34 (5)	13 (4)
Not stated	3 (0)	1 (0)	Not stated	15 (2)	13 (4)
**Country of birth**			Gluten intolerance	9 (1)	3 (1)
Sweden	514 (83)	316 (92)	Not stated	9 (1)	14 (4)
Other country	107 (17)	28 (8)	Pelvic organ prolapse	13 (2)	13 (4)
Not stated	2 (0)	0 (0)	Not stated	17 (3)	12 (4)
**Place of residency**			Rheumatism	40 (6)	19 (6)
Rural district	58 (9)	34 (10)	Not stated	13 (2)	3 (1)
Village/Small town	193 (31)	93 (27)	Kidney disease	19 (3)	8 (2)
> 500.000 citizens	371 (60)	214 (62)	Not stated	13 (2)	3 (1)
Not stated	1 (0)	3 (1)	Lung disease	37 (6)	12 (4)
**Smoking**			Not stated	13 (2)	3 (1)
Current smoker	143 (23)	88 (26)	Thrombosis	46 (7)	16 (5)
Former smoker	191 (31)	108 (31)	Not stated	13 (2)	3 (1)
Never smoker	281 (45)	147 (43)	Osteoporosis	59 (9)	25 (7)
Not stated	8 (1)	1 (0)	Not stated	13 (2)	3 (1)
**Body Mass Index**			Psychological disorders	79 (13)	43 (13)
≤18.5	17 (3)	5 (2)	Not stated	13 (2)	3 (1)
18.5–25	270 (43)	163 (47)	Neurological disorders	15 (2)	3 (1)
25–30	199 (32)	116 (34)	Not stated	13 (2)	3 (1)
≥30	99 (16)	43 (13)	Joint disorder	171 (27)	95 (28)
Not stated	38 (6)	17 (5)	Not stated	13 (2)	3 (1)
**Exercise**			**Medication**		
Never	72 (12)	20 (6)	Using any kind of medication	440 (71)	194 (56)
At least once a month	84 (13)	59 (17)	Not stated	8 (1)	6 (2)
At least once a week	450 (72)	262 (76)	Estrogen	226 (36)	50 (15)
Not stated	17 (3)	3 (1)	Not stated	10 (2)	5 (2)
**Parity**			**Diagnosis**		
Never given birth	156 (25)	45 (13)	Sarcoma uteri	30 (5)	
1–3 Children	418 (67)	280 (81)	Vulvar cancer	6 (1)	
> 3 Children	49 (8)	19 (6)	Vaginal cancer	14 (2)	
Not stated	0 (0)	0 (0)	Cervical cancer	146 (23)	
**Delivery**^£^			Endometrial cancer	363 (58)	
Fast (< 5h)	252 (40)	147 (43)	Ovarian cancer	50 (8)	
Slow (> 24 h)	139 (22)	88 (26)	Fallopian tube cancer	14 (2)	
Vacuum	41 (7)	43 (13)	Not stated	0 (0)	
Forceps	11 (2)	7 (2)	**Treatment modality**		
Episiotomy	133 (21)	117 (34)	Surgery + EBRT^‡^	47 (8)	
Caesarean	28 (4)	40 (12)	Surgery + EBRT^‡^ + BT^¥^	338 (54)	
Breech birth	19 (3)	20 (6)	Surgery + EBRT^‡^ + Chemo^#^	64 (10)	
Not stated^Ω^	7 (1)	2 (1)	Surgery + EBRT^‡^ + BT^¥^ + Chemo^#^	113 (18)	
**Child weight at delivery**			EBRT^‡^	2 (0)	
> 4 kg, 1 child	79 (13)	54 (16)	EBRT^‡^ + BT^¥^	27 (4)	
> 4 kg, ≥ 2 children	44 (7)	29 (8)	EBRT^‡^ + Chemo^#^	8 (1)	
Not stated	4 (1)	261 (76)	EBRT^‡^ + BT^¥^ + Chemo^#^	23 (4)	
**Anal injury**^₸^^,^^£^			Not stated	1 (0)	
Yes	18 (3)	18 (5)			
Not stated	19 (3)	2 (1)			

*Approximate age at follow up. Calculated as 2006 –year of birth.

^£^Only dichotomous variables in this category. The numbers of negative values are left out.

^Ω^Number of survivors for which no information was recorded regarding the delivery variables.

^₸^Injury inflicted during delivery or at other occasion. IBS^₽^ denotes Irritable Bowel Syndrome.

EBRT^‡^ denotes External Beam Radiation Therapy. BT^¥^ denotes Brachy Therapy. Chemo^#^ denotes Chemotherapy

### Number of factors

**Fig 1:**
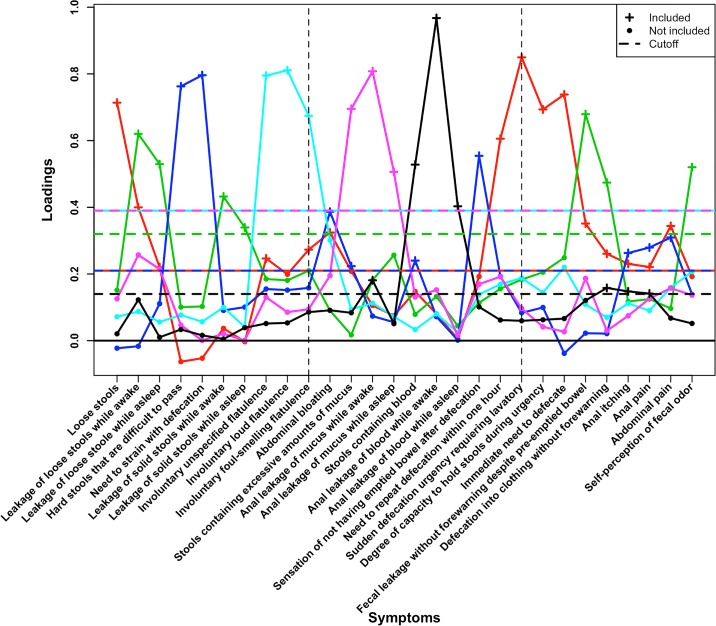
The estimated factor loadings onto the six factors after the Varimax rotation was performed. Factor loadings are colored according to factor affiliation and are connected by solid lines of the corresponding color. Dashed horizontal lines correspond to the factor specific cutoffs suggested by the Variable Cutoff Method. Crosses of a specific color correspond to factor loadings strictly greater in magnitude than the cutoff of the same color whereas solid dots of a specific color correspond to factor loadings smaller in magnitude than the cutoff of the corresponding color. Using the Variable Cutoff Method 10000 parametric bootstrap estimates of the factor loadings were calculated and 0,0.01,…,0.99,1 were used as candidate cutoffs.

The optimal number of factors was estimated to be six both when we used Parallel analysis and a bootstrap version of Kaiser's rule (Fig 1 and data in statistical appendix S1 File) [23].

### Symptoms included in each factor

**Fig 2:**
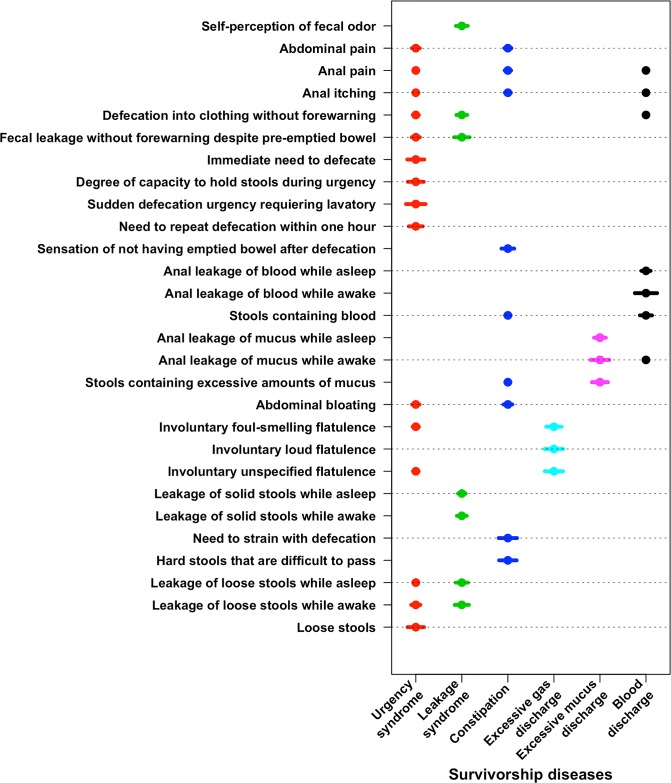
The result of applying the cutoffs suggested by the Variable Cutoff Method to the estimated factor loadings onto the six factors. Dots correspond to factor loadings that are strictly greater in magnitude than the factor specific cutoff. Lines through the dots correspond to the magnitude of the specific factor loadings and are presented for comparison purposes only with the aim of identifying the variables that most heavily load onto a specific factor and thus to aid interpretation. The plot illustrates how cutoffs on factor loadings ease the interpretation of the factor loading structure produced by EFA. Several factor loadings are discarded by the Variable cutoff method. Based on this reduced factor loading structures the six factors were interpreted as: Urgency syndrome (red), Leakage syndrome (green), Constipation (dark blue), Excessive gas discharge (light blue), Excessive mucus discharge (magenta), Blood discharge (black).

**Fig 3:**
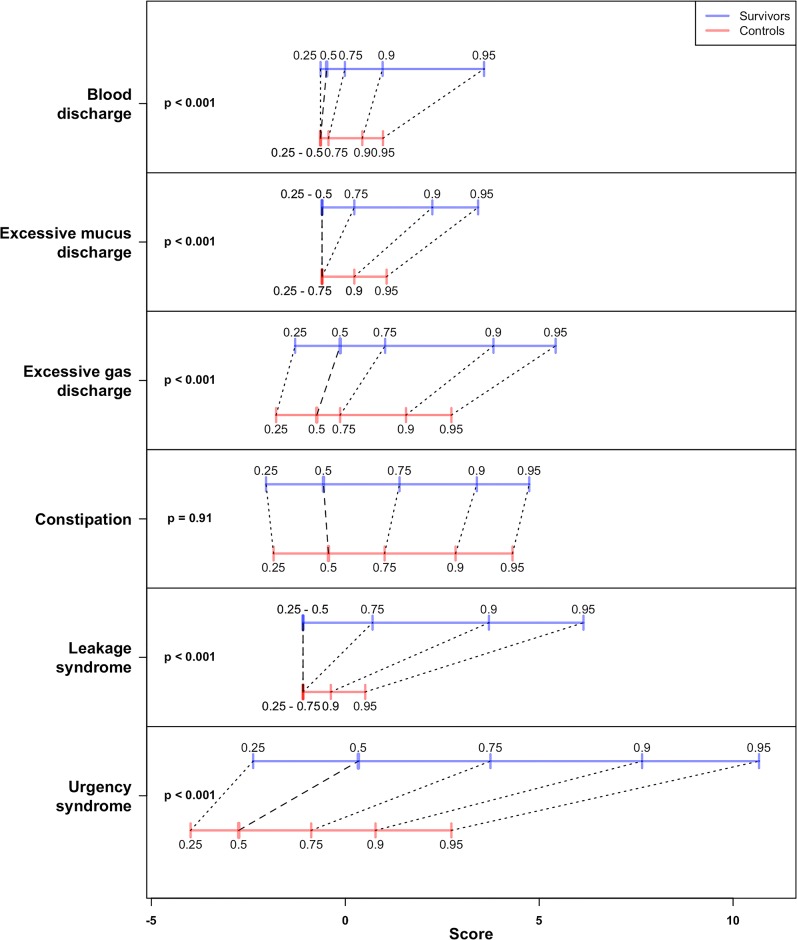
Comparisons between estimated factor score quantile positions of survivors and controls for the six factors. The 0.25, 0.5 (median), 0.75, 0.9 and 0.95 sample quantiles are presented. Scores were calculated based on the reduced factor loading structure. Prior to calculating scores a simple mode imputation was performed. Further Mann-Whitney p-values were calculated and are presented to the left in the figure. Except in the case of the constipation factor, the factor scores of the treated population were found to be distributed significantly differently from the scores of the non-treated population. Clearly, in all cases where these distributions differ, the scores of the survivors tend to be larger than the scores of the controls.

In Table 2 the varying cut-offs for the factor loadings across the six factors are presented along with the factor loading for each symptom and factor. Since each factor is regarded as a syndrome that may reflect a distinct survivorship disease (with the long-term symptoms being seen as manifestations of the disease), we in Table 2 describe the factors as *urgency syndrome*, *leakage syndrome*, *constipation*, *excessive gas discharge*, *excessive mucus discharge* and *blood discharge*. The factors in Table 2 and Figs 2 and 3 are ordered according to the proportion of variance in the data that is explained. Fig 2 shows a graphical view of the symptoms having factor loadings above the factor-specific factor-loading cutoffs. The bars represent the value of the factor loading (and do not represent a confidence interval)–the broader the bar is, the greater the proportion of the variance of the symptom the specific factor explains. Thirteen of the 28 symptoms loaded onto two or more factors.

**Table 2:** Estimated factor loading structure and factor model properties.

SYMPTOM	INCIDENCE		ESTIMATED FACTOR LOADINGS^¥^					
	Number of events/Total number of individuals¤		URGENCY SYNDROME	LEAKAGE SYNDROME	CONSTIPATION	EXCESSIVE GAS DISCHARGE	EXCESSIVE MUCUS DISCHARGE	BLOOD DISCHARGE
	Controls	Survivors	Cutoff^†^					
	(%)	(%)	0.21	0.32	0.21	0.39	0.39	0.14
	82/344	308/616						
Loose stools*	(24)	(50)	**0.71**	0.15	-0.02	0.07	0.13	0.02
Leakage of loose stools	3/344	52/620						
while awake*	(1)	(8)	**0.40**	**0.62**	-0.02	0.09	0.26	0.12
Leakage of loose stools	3/343	13/623						
while asleep*	(1)	(2)	**0.22**	**0.53**	0.11	0.06	0.21	0.01
Hard stools that are difficult to	48/343	75/619						
pass*	(14)	(12)	-0.06	0.10	**0.76**	0.08	0.05	0.03
Need to strain with defecation	28/337	54/615						
at least half of the time	(8)	(9)	-0.05	0.10	**0.80**	0.06	0.00	0.02
Leakage of solid stools	1/344	11/620						
while awake*	(0)	(2)	0.04	**0.43**	0.09	0.10	0.02	0.00
Leakage of solid stools	0/344	4/623						
while asleep*	(0)	(1)	0.00	**0.34**	0.10	0.04	0.00	0.04
Involuntary unspecified	48/343	166/620						
flatulence*	(14)	(27)	**0.25**	0.18	0.15	**0.79**	0.13	0.05
	33/344	134/621						
Involuntary loud flatulence*	(10)	(22)	0.20	0.18	0.15	**0.81**	0.09	0.05
Involuntary foul-smelling	33/343	156/618						
flatulence*	(10)	(25)	**0.27**	0.21	0.16	**0.67**	0.09	0.09
	104/342	198/617						
Abdominal bloating*	(30)	(32)	**0.32**	0.09	**0.39**	0.30	0.20	0.09
Stools containing excessive	9/343	66/618						
amounts of mucus *	(3)	(11)	0.21	0.02	**0.22**	0.09	**0.70**	0.08
Anal leakage of mucus	5/343	31/618						
while awake*	(1)	(5)	0.11	0.18	0.07	0.11	**0.81**	**0.18**
Anal leakage of mucus	1/344	7/620						
while asleep*	(0)	(1)	0.07	0.26	0.06	0.07	**0.51**	0.05
Stools containing excessive	6/341	27/617						
amounts of blood*	(2)	(4)	0.15	0.08	**0.24**	0.03	0.13	**0.53**
Anal leakage of blood	1/343	12/620						
while awake*	(0)	(2)	0.08	0.13	0.07	0.08	0.15	**0.97**
Anal leakage of blood	0/343	0/621						
while asleep*	(0)	(0)	0.00	0.04	0.00	0.02	0.02	**0.40**
Sensation of not having emptied	39/340	110/619						
after defecation*	(11)	(18)	0.19	0.11	**0.55**	0.14	0.17	0.10
Need to repeat defecation within	32/341	204/619						
one hour*	(9)	(33)	**0.61**	0.16	0.19	0.17	0.19	0.06
Sudden defecation urgency	33/341	245/616						
requiring lavatory*	(10)	(40)	**0.85**	0.18	0.08	0.19	0.10	0.06
Inability to hold stools for >5	40/344	269/615						
minutes during urgency‡	(12)	(44)	**0.69**	0.21	0.10	0.14	0.04	0.06
	17/341	146/620						
Immediate need to defecate*	(5)	(24)	**0.74**	0.25	-0.04	0.22	0.03	0.07
Fecal leakage without warning	3/344	52/620						
despite previous defecation*	(1)	(8)	**0.35**	**0.68**	0.02	0.11	0.19	0.12
Defecation into clothing without	3/344	18/621						
forewarning*	(1)	(3)	**0.26**	**0.47**	0.02	0.07	0.03	**0.16**
	29/343	56/621						
Anal itching*	(8)	(9)	**0.23**	0.12	**0.26**	0.11	0.07	**0.15**
	12/343	32/621						
Anal pain*	(3)	(5)	**0.22**	0.12	**0.28**	0.09	0.13	**0.14**
	28/340	112/616						
Abdominal pain*	(8)	(18)	**0.34**	0.10	**0.31**	0.16	0.16	0.07
	5/340	21/619						
Self-perception of fecal odor*	(1)	(3)	0.19	**0.52**	0.14	0.21	0.14	0.05
			**ESTIMATED FACTOR MODEL PROPERTIES**					
Sum of squares of loadings			3.7	2.4	2.2	2.1	1.8	1.6
Proportion of variance explained			0.13	0.09	0.08	0.08	0.06	0.06
Cumulative proportion of variance explained#			0.13	0.22	0.3	0.38	0.44	0.5

^¥^Estimated factor loadings greater than the corresponding cutoff produced by the Variable Cutoff Method are presented in bold font.

^¤^The denominators deviate from 344 (the number of controls) and 623 (the number of survivors) due to missing values.

^†^Cutoffs on estimated factor loadings produced by the Variable Cutoff Method.

*At least once a month.

^**+**^In the Factor analysis all categories (“No, not at any occasion”, “Yes, more seldom than at half of the occasions when I have defecated”, “Yes, more often than at half of the occasions”, “Yes, at every occasion”) were used. Cutoff used for frequencies in the second and third columns.

^‡^In the Factor analysis all categories (”Not appropriate, I have not had any urgency”,”Shorter than 1 minute”,”Between 1 and 5 minutes”,”Between 1 and 30 minutes”,”30 minutes or longer”) were used. Cutoff used for frequencies in the second and third columns.

^**#**^Cumulative sum of the proportions of variance explained. The cumulative sum of a vector v = (a1, a2, a3,…, an) is defined as v’: = (a1, a1+ a2, a1+ a2+ a3,…, a1 + a2+ a3 +…+ an)

### Comparison between survivors and controls

Fig 3 shows that being a survivor is associated with five of the factors but not with the factor cited as *constipation*. Notwithstanding that, the symptoms loading onto this factor may have different causes in survivors and population-based controls, we thus have no data to support the belief that *constipation* is a syndrome related to a survivorship disease.

Classifying a survivor as having a specific syndrome with a factor loading above that of the 95 percentile among the controls, we obtained the following numbers: *urgency syndrome* (190 of 623, 30 percent), *leakage syndrome* (164 of 623, 26 percent), *excessive gas discharge* (93 of 623, 15 percent), *excessive mucus discharge* (102 of 623, 16 percent) and *blood discharge* (63 of 623, 10 percent).

## Discussion

Using a population-based setting for identifying and retrieving patient-reported information from cancer survivors, we previously have found that gynecological-cancer survivors three to 15 years after radiotherapy have 28 different gastrointestinal symptoms [14]. Applying a modified factor analysis, and comparing with population controls, our results indicate that these 28 symptoms may be seen as five syndromes that may be manifestations of five different radiation-induced survivorship diseases causing a decrease in bowel health.

Technically speaking, the first step in the analyses we performed answered the question “How many factors can be disentangled [20]? In the second step we sought to determine which estimated factor loadings reflect non-zero population factor loadings. The initial identification of six factors was done with two different methods [21]. This observation is thus robust. However, a number of cautions indicate that six may not be the exact number in real life. Although we, during a meticulous qualitative phase, interviewed survivors and experts to search for all manifestations of survivorship diseases occurring among gynecological cancer survivors, we may have missed key symptoms. Moreover, despite repeated face-to-face validation we may have missed key variations in the wording used to identify each of the different symptoms; this error may turn up as varying degrees of sensitivity and specificity in identifying different symptoms during the data collection. All these weaknesses may have compromised our ability to disentangle additional factors that may be captured in a new data collection.

In comparing factor-score quantiles of the cancer survivors and population controls, we found a statistically significant difference for five of the six identified factors. Thus, in the application of these statistical parameters to the real world, we consider the five factors as five distinct treatment-induced survivorship syndromes. We have no biological data from the survivors; for example, we have no tests in the blood or feces for markers indicating inflammatory processes or fibrosis. We are not aware of any previous effort similar to ours in identifying survivorship syndromes. Nevertheless, based on biological, physiological and medical facts presented in the S1 Table we label the five syndromes *leakage syndrome*, *urgency syndrome*, *excessive mucus discharge*, *excessive gas discharge* and *blood discharge*. The initiation of the survivorship diseases, manifested by these syndromes, results when unwanted ionizing radiation reaches the anal-sphincter region, the rectum, the sigmoid, the small bowel and possibly also other volumes of normal tissue in the pelvic cavity (S2 Fig). These disease labels are preliminary as are the designations of the organs involved and the mechanisms.

When we varied the details of the factor analysis, fine-tuning which variables to include in a certain factor, e.g., by making varying assumptions for the determination of cut-offs, we produced somewhat different results (data not shown). In the interpretation we thus have a variation in the degree of evidence for which symptoms to include in one of the identified syndromes. But some clear distinctions can be made. No analysis, for example, produced a result in which the three symptoms included in *excessive mucus discharge* loaded in the factor interpreted as *urgency syndrome*. Results thus clearly indicate these mucus-related symptoms are produced by processes other than the processes giving the symptoms in *urgency syndrome*.

Concerning the pathophysiology, we know little about the processes that ultimately produced the symptoms included in respective syndrome. Probably endothelial damage in capillaries in the gut wall attracts white blood cells; they may aggregate causing hypoxia and later ischemia. We believe inflammatory and fibrotic processes in the gut wall, as well as stem-cell depletion, play a role. Possibly changes in the wall of the small bowel, proximal colon, distal colon (sigmoid colon) and rectum are related to varying symptoms. *Excessive gas discharge* and *excessive mucus discharge* probably are related to a changed composition and function of the gut microbiota, and we know the microbiota interacts with the gut wall. Endoscopically, in survivors with *blood discharge*, one can inspect a gut wall exclusively comprised of connective tissue, with telangiectasia and ulcerations that bleed on the surface. Fibrotic muscle in the internal and external anal sphincters probably explains leakage-related symptoms, but some symptoms in the *leakage syndrome* may also reflect increased pressure on the sphincters. Gall salt malabsorption, and bacterial overgrowth from the colon to the small bowel are well recognized clinically but we have no data to relate these phenomena to the syndromes we identified. If a relation exists between age and symptom occurrence among the population-based controls, it is weak at most. That the controls are younger than the cancer survivors probably does not spuriously affect the conclusion that one of the six identified syndromes is not radiation-induced.

Lacking a basic understanding of how different pathophysiological processes in different parts of the abdomen and gut can be linked to the syndromes we identified (or to single symptoms), it is too early to suggest a nosology for the survivorship diseases decreasing bowel health. This study only concerns females; in males (prostate-cancer survivors) a corresponding factor analysis resulted in four syndromes that may be cited as *leakage syndrome*, *urgency syndrome*, *excessive mucus discharge* and *excessive gas discharge/abdominal pain* [24]. That is, in that population, four of the five syndromes we identified were disentangled and the data indicate the same survivorship diseases affect men and women concerning the bowel. Possibly new factor analyses on already collected material can give us more information. Combining the loading factor for each symptom with symptom frequency we get a metric (factor score) of the intensity of the syndrome. Using this metric we can investigate different effects of the five syndromes on factors such as dose to different risk organs, smoking, time since treatment and diet—investigating whether the syndromes may manifest different pathophysiological processes. Such a metric of the intensity of a syndrome may assist the search for preventive measures. Toxicity scores and quality-of-life instruments may introduce a noise when symptoms from different syndromes are combined. Such instruments may mix symptoms from different syndromes that reflect different pathophysiological processes. If for example, a genetic factor, or dose to a specific risk organ, is studied, the noise may be reduced if symptoms related to the effect (a pathophysiological process) by the gene or the dose can be studied separately. Concerning today’s survivors, time will show if we can get sharper diagnostic tools and better treatments if we combine the identification of syndromes with tests in the blood and feces, and possibly also endoscopic studies and X-rays, to specific survivorship diseases for which we develop prevention, alleviation and treatments.

## Supporting information

S1 Fig Flow chart. Inclusion an exclusion criteria of the gynecological-cancer survivors and matched population-based controls and questionnaire return rate.
(PDF)

S2 Fig Preliminary designations of the organs involved in the survivorship diseases. (TIF)

S1 File Statistical appendix. Modified Exploratory Factor Analysis to define the number of factors that best describe the correlation matrix of the data, factor loadings, factor-specific cutoffs for factor loadings and factor scores.
(DOCX)

S1 Table Information regarding survivorship diseases and references. Possible radiation-induced pathophysiological processes in the suggested survivorship diseases.
(DOCX)
