# Supplementary material for: Identifying radiation-induced survivorship syndromes affecting bowel health in a cohort of gynecological cancer survivors
Source: PLoS One. 2017 Feb 3;12(2):e0171461. doi: 10.1371/journal.pone.0171461 (PMC5291512; doi:10.1371/journal.pone.0171461)
Supplement: S1 Fig — Inclusion an exclusion criteria of the gynecological-cancer survivors and matched population-based controls and questionnaire return rate. (PDF) [file pone.0171461.s001.pdf]

Survivors

1800 subjects, treated for gynaecological cancers with external pelvic radiotherapy at Karolinska University Hospital, Stockholm, or Sahlgrenska University Hospital, Gothenburg, between the years 1991-2003.

Controls

486 control women from the Swedish Population Registry, matched for age and residential area

977 did not meet the eligibility criteria	***Number***
Deceased at follow-up	497
Born before 1927	436
Could not understand/read Swedish	23
Had recurrence	19
Had not received pelvic radiotherapy	2

8 did not meet the eligibility criteria	***Number***
Could not understand/read Swedish	5
Had received pelvic radiotherapy	3

An introduction letter was sent to 823 eligible survivors

An introduction letter was sent to 478 eligible controls

Reasons for non-participation:	***Number***
No reason provided	29
Physical reason	21
Not reachable	17
Psychosocial reason	14
Psychological reason	9
Family members said no	2

Reasons for non-participation:	***Number***
No reason provided	37
Physical reason	13
Not reachable	5
Psychosocial reason	3

731 (89%) survivors gave informed oral consent and was sent a questionnaire

420 (88%) controls gave informed oral consent and was sent a questionnaire

81 survivors did not complete the study	***Number***
Agreed but did not return questionnaire	52
Sent back an empty questionnaire	29

76 controls did not complete the study	***Number***
Agreed but did not return questionnaire	66
Sent back an empty questionnaire	10

650 (79%) survivors returned a completed questionnaire and participated in the project

344 (72%) controls returned a completed questionnaire and participated in the project

27 survivors were excluded after participation	***Number***
Had undergone an ostomy	20
At least 30% missing values across the studied symptoms	7

0 controls were excluded after participation

Survivors

623 (76%) survivors were included in the data analysis

Controls

344 (72%) controls were included in the data analysis
