# Supplementary figures and images for: Identifying radiation-induced survivorship syndromes affecting bowel health in a cohort of gynecological cancer survivors
Source: PLoS One. 2017 Feb 3;12(2):e0171461. doi: 10.1371/journal.pone.0171461 (PMC5291512; doi:10.1371/journal.pone.0171461)

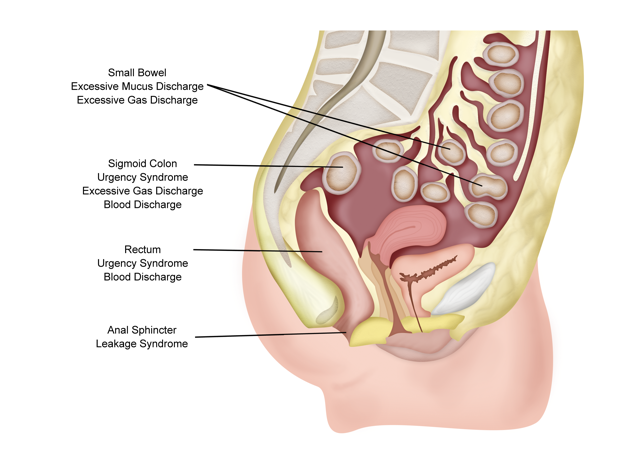

Supplement: S2 Fig — (TIF) [file pone.0171461.s002.tif]
