# Supplementary material for: Identifying radiation-induced survivorship syndromes affecting bowel health in a cohort of gynecological cancer survivors
Source: PLoS One. 2017 Feb 3;12(2):e0171461. doi: 10.1371/journal.pone.0171461 (PMC5291512; doi:10.1371/journal.pone.0171461)
Supplement: S1 File — Modified Exploratory Factor Analysis to define the number of factors that best describe the correlation matrix of the data, factor loadings, factor-specific cutoffs for factor loadings and factor scores. (DOCX) [file pone.0171461.s003.docx]

1 Appendix

**1.1 Preliminary considerations.**

In this article the data were modelled using Exploratory Factor Analysis (EFA) as implemented by the fa() function in the R library *psych*.1 All calculations were performed after the data were subjected to two preliminary filtrations. First, survivors were excluded if they have stomi. This led to the exclusion of 20 survivors. Second, survivors were excluded if more than 30 percent of the data for the 28 variables studied was missing. This criterion resulted in seven survivors being excluded. After the two filtrations 623 survivors remained.

Since ordinal data were studied, Spearman correlations were consistently used as input to the EFA. When estimating correlations between variables, pairwise deletion of missing data was employed. For example, given two samples $V_{1}=\left( v_{11},NA,v_{13},v_{14} \right)$ and $V_{2}=\left( v_{21},NA,v_{23},NA \right)$ of random variables $V_{1}$ and $V_{2}$ the correlation between $V_{1}$ and $V_{2}$would be estimated based on the reduced samples $\underline{V}_{1}= \left( v_{11},v_{13} \right)$and $\underline{V}_{2}=\left( v_{21}, v_{23} \right)$. EFA parameter estimates were consistently found using Maximum Likelihood Estimation (MLE). Once again, since ordinal data were studied the assumption of multivariate normality underlying MLE was violated. However, the literature suggests that MLE works well in the context of EFA in spite of this violation.2

In order to assess whether the data studied were suitable for EFA Bartlett’s test of sphericity was performed on the sample correlation matrix and the KMO (Kaiser-Meyer-Olkin) measure of sampling adequacy (MSA) was then calculated, once again from the sample correlation matrix. Bartlett’s test resulted in a P value $<0.001$ and the test was thus considered significant. The MSA equaled 0.88. Since the KMO criterion states that the MSA should be greater than 0.5, the data were judged suitable for EFA.

Two central problems to be faced when using EFA are first the making of an informed decision on how many factors to use in the model and second how to increase the interpretability of the EFA estimates. These problems were addressed as follows.

**1.2 Assessing the number of factors to use.**

In order to assess the appropriate number of factors to use in the EFA model of the data *parallel analysis* and a non-parametric bootstrap version of *Kaiser’s rule* were used.3–5 The parallel analysis was based on 10.000 randomly permuted versions of the data and 99 percent quantile confidence intervals. Likewise, the variation of Kaiser’s rule was based on 10.000 non- parametric bootstrap estimates. In the Kaiser’s rule case the choice was made to be conservative and therefore accept no bootstrap estimates below 1. As seen in figure 1 both methods prescribe using six factors.


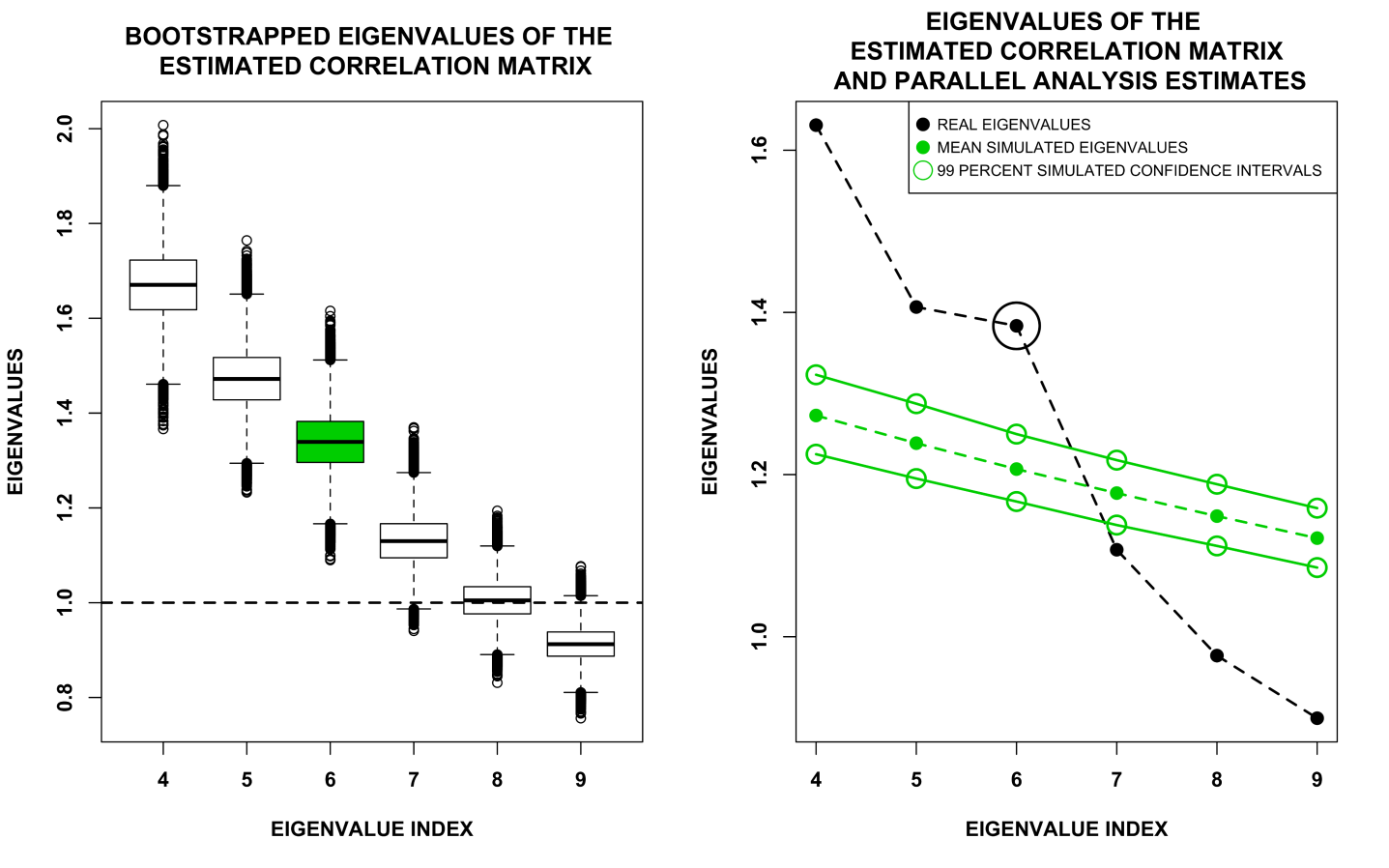


**Fig 1.** Determining the number of factors to use in an EFA model of the data. Left panel: A non-parametric bootstrap version of Kaiser’s rule. The rule prescribes using as many factors as there are eigenvalues of the data correlation matrix greater than 1. Shown here are box plots of 10.000 non-parametric bootstrap estimates of the ordered eigenvalues of the data correlation matrix. Right panel: Parallel analysis. Parallel analysis prescribes using as many factors as there are eigenvalues of the data correlation matrix strictly above the $\alpha$-percent confidence intervals based on the ordered eigenvalues of the correlation matrices of $N$ randomly perturbed versions of the data. In our case $N=10.000$ and $\alpha=99$ percent. Both methods prescribe using six factors.

**1.3 Increasing the interpretability of the EFA estimates.**

The first step in increasing the interpretability of the EFA estimates is to rotate the initial solution, a process that is eligible since orthogonal rotations respect EFA model fit. In this study the Varimax rotation was consistently used; this rotation maximizes the sum across the factors of the within factor variances of the squares of the factor loadings under the constraint of orthogonality.6 However, in order to further increase the interpretability of the EFA estimates several methods exist that aim at assessing what factor loadings can be considered significant. A crude such method is simply setting a cutoff of 0.3 on rotated factor loadings. We describe below an improved version of this method, which sets a uniform cutoff on factor loadings per factor based on parametric bootstrapping. In order to assess the performance of this method a simulation study was undertaken. The method and the results from the simulation study are described in sections 1.5 - 1.5.1 below.

**1.4 Comparing factor scores among survivors and population controls.**

After applying the Variable Cutoff Method to the estimated factor loading structure, factor scores were calculated. That is, the factor scores were calculated based on the relatively sparse factor loading matrix obtained by substituting 0 for estimated factor loadings smaller in absolute value than the factor specific cutoffs produced by the Variable Cutoff Method. In order to obtain a meaningful comparison of the factor scores among survivors and population controls, the separate data sets were imputed using mode imputation of the variables after which the data sets were combined into one data set which was standardized i.e. rescaled to have mean 0 and marginal variances of 1. The imputations were necessary in order to be able to calculate factor scores for all individuals. After standardization, factor scores were calculated as linear combinations of the values of an individual across the 28 symptoms studied and the loading factors corresponding to a specific factor. More specifically, if $L_{i}=\left( l_{1i},\ldots,l_{28i} \right)$ were the loading factors corresponding to factor $i$ and the values of individual $j$ across the 28 symptoms were $x_{j}= \left( x_{j1},\ldots, x_{j28} \right)$, then the score of individual j on factor i was calculated as:

$$s_{ji}= \left( x_{j1}l_{1i}+\ldots+x_{j28}l_{28i} \right)$$

After the calculation of the factor scores of all individuals for all factors, comparisons of the scores among survivors and population controls were performed using the Mann-Whitney *U* test. The six tests resulted in P values $<0.001$ in five cases and a P value of 0.91 in one case.

**1.5 Simulation study.**

Nine distributions of known factor structures were studied. These factor structures are depicted in figure 2. Six 16-dimensional and three 30- dimensional distributions were studied. The former distributions were generated using four factors and the latter using six factors. As seen in figure 2, the factor loading structures were varied with respect to maximum, minimum and mean factor loadings as well as with respect to the within factor factor loading spread and the amount of overlap between factors.

For each of the nine distributions the following steps were undertaken:

1.Generate 623 (the number of respondents studied) samples from the present distribution with factor loading structure $L$, where factor loadings on a specific factor are represented by the columns of$L$.

2.Estimate the Spearman correlation of the generated data, fit an EFA model and rotate the solution using the Varimax rotation. Call the estimated factor loading matrix $L^{'}$.

3.Parametric bootstrap: Carry out for 2500 times the generation of 623 samples from a distribution with factor structure $L^{'}$. For each sample, estimate the Spearman correlation, and fit an EFA model generating factor loading matrix estimates $L_{n}^{''}$ , where $n=1,\ldots, 2500.$ Rotate each $L_{n}^{''}$ orthogonally to maximally resemble $L^{'}$ using Procrustes rotations, thus generating $L_{n}^{'''}$, where $n=1,\ldots, 2500.$

4.Now consider $C=\left( 0, 0.01, \ldots, 0.99,1 \right)$ as candidate cutoff-values on factor loadings and study factor number $m$. Define matrix $M$ as $M_{i\cdot}=L_{\cdot i}^{'''}$. $M$ thus contains as column $i$ the bootstrapped factor loadings of variable $i$ onto factor $m$. For each candidate cutoff-value $c$ define the two sets of indices:

- $PZ :=\left\{ i|\exists j,k such that \left( M_{ji}>c \right)\wedge\left( M_{ki}<c \right) \right\}$.
- $PNZ := {PZ}^{c}$.

Here $PZ$ stands for Potential Zeros and $PNZ$ stands for Potential Non Zeros. Further evaluate the following function:

$$COST\left( c \right):=W_{z}\sum_{i\in PZ} \#(|M_{\cdot i}|>c)+ W_{nz}\sum_{i\in PNZ} \#(|M_{\cdot i}|\leq c)$$

where

$$W_{z}=\frac{w}{|PZ|},W_{nz}= \frac{1-w}{|PNZ|},w\in\left[ 0,1 \right]$$

and find the $c_{m}^{'}\in C$ that minimizes $COST$. Use this value as a cutoff on $L_{\cdot m}^{'}$ and record which factor loadings are set to zero and which are retained.

5.Repeat 4 for all $m$.

6.Estimate the true positivity, false positivity and false discovery rates by comparing actual zero and non-zero loadings in the underlying distribution to the predicted zeros and non-zeros after applying the $c_{\cdot}^{'}$s.

7.Store results and repeat steps 1-6 30 times.

The $COST$ function measures the extent to which the bootstrap estimates of the potential zero factor loadings are contained within $\pm$ a candidate cutoff (first sum) and the extent to which the bootstrap estimates of the potential non-zero factor loadings are situated outside $\pm$ a candidate cutoff (second sum). The weights $W_{z}$ and $W_{nz}$ are constructed in such a way that $W_{z}\left| PZ \right|+W_{nz}\left| PNZ \right|= 1$ and the internal parameter w determines the ex- tent to which potential zeros and potential non zeros contribute to the cost. The dependency on the number of potential zeros and potential non zeros of the weights further has the additional effect of increasing the marginal cost of expanding the cutoff outwards when there are few non zeros, i.e. when the candidate cutoff is large, and conversely increases the incentive for containing potential zeros when to cutoff is small.

The performance of the above method, referred to below as the Variable Cutoff Method, when applied to the nine distributions in figure 2 is depicted in figures 3, 4 and 5.

**
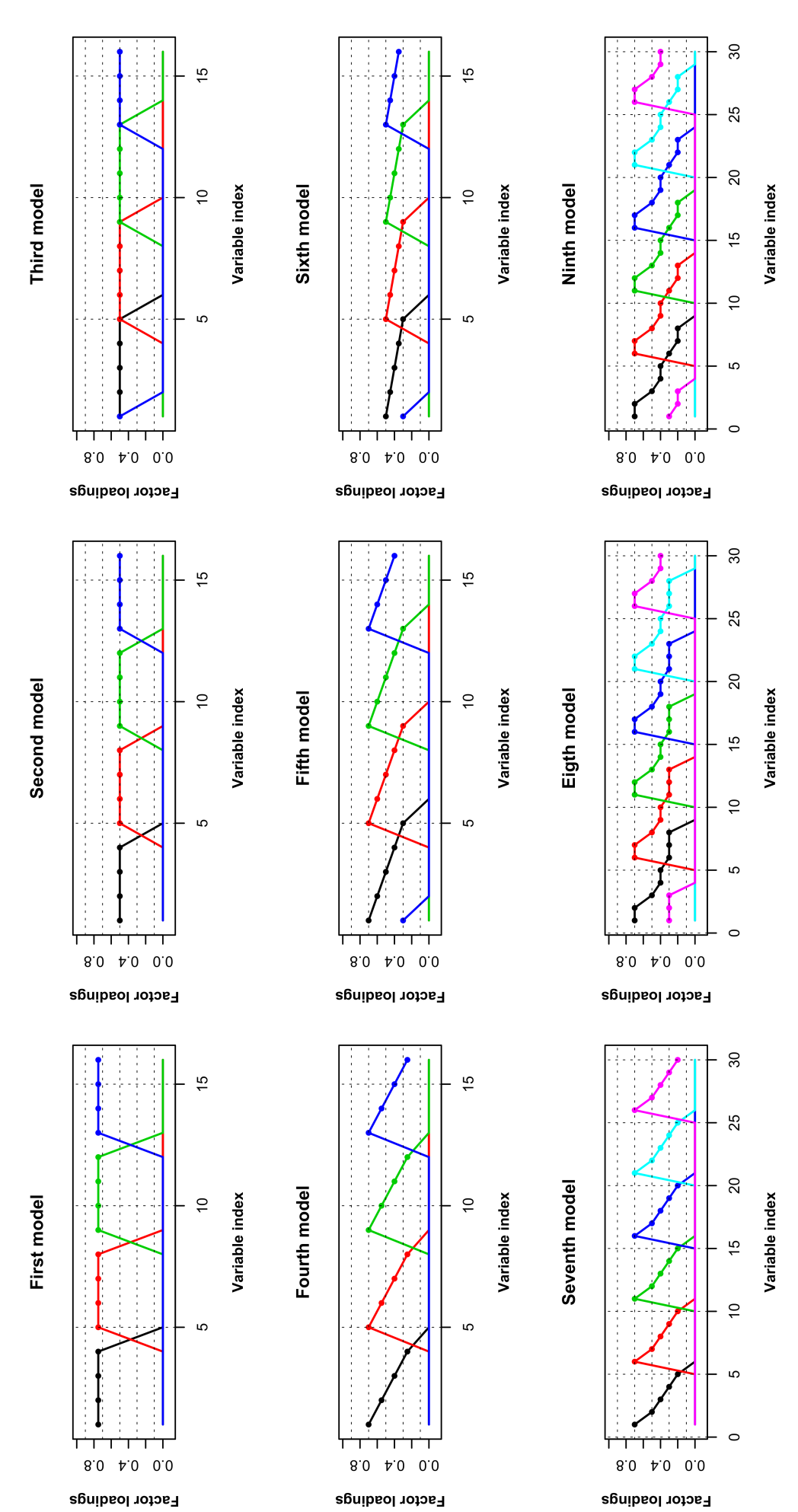
**

**Fig 2.** Factor loading structures of the distributions included in the simulation study. Factor loadings are colored according to factor index.

**1.5.1 Simulation study results.**

The performance of the Variable Cutoff Method was evaluated by applying it to nine distributions of known factor structures. In the known factor structures several factor loadings are exactly zero and the aim of the method could thus be described as discriminating between zero and non-zero factor loadings. The Variable Cutoff Method was thus evaluated as a binary dis- crimination method, recording the true positivity, false positivity and false discovery rates when applied to the nine distributions. Furthermore, the same measures were recorded using a constant 0.3 cutoff. The objective was to produce a true discovery rate with a value as high as possible while keeping the false positivity and false discovery rate at acceptable levels - more precisely the goal was to produce a higher true positivity rate than when using a 0.3 cutoff under the same constraints. To this end the tuning parameter $w$ of the $COST$function was varied and the above measures were recorded. As seen in figures 3-5, using $w=0.7$ consistently produced mean false discovery rates below 0.05 and mean false positivity rates below 0.025 while producing mean true positivity rates higher than those produced using a constant cutoff of 0.3 except in the cases of models 2 and 3. How- ever, these two models are trivial cases in which the 0.3 cutoff happens to work perfectly and the variable cutoff method produces mean true positivity rates very close to perfect. Looking at the more complex models, especially models 7-9, where models 8-9 most closely resemble the real world data studied, the variable cutoff method produces mean true positivity rates that are substantially higher than the ones produced using the constant 0.3 cut- off. Thus, the simulation study undertaken suggests that using the variable cutoff method with $w=0.7$ allows us to extract more relevant information from the estimated factor loadings then does the use of a constant 0.3 cutoff while keeping the level of false discoveries at an acceptable level.

An interesting result from the simulation is also, however, that the 0.3 consistently produces perfect false positivity and false discovery rates. Thus, if perfect performance in these measures is of utmost importance, this cutoff is a good choice. Of course, this then comes at the price of a lower true positivity rate than offered by alternative methods.

Of course there are other possible methods for filtering away noise loadings that are higher in resolution, for example P value based methods that assign a P value to each factor loading. However, we found when using such methods that estimated and expected rejection rates differed, in sign and magnitude, to an extent that did not allow us reliably interpret the results when applying these methods to our data. Normal, percentile and BCa bootstrap P values were used and their behaviors were evaluated when applied to the nine distributions described in figure 2.7


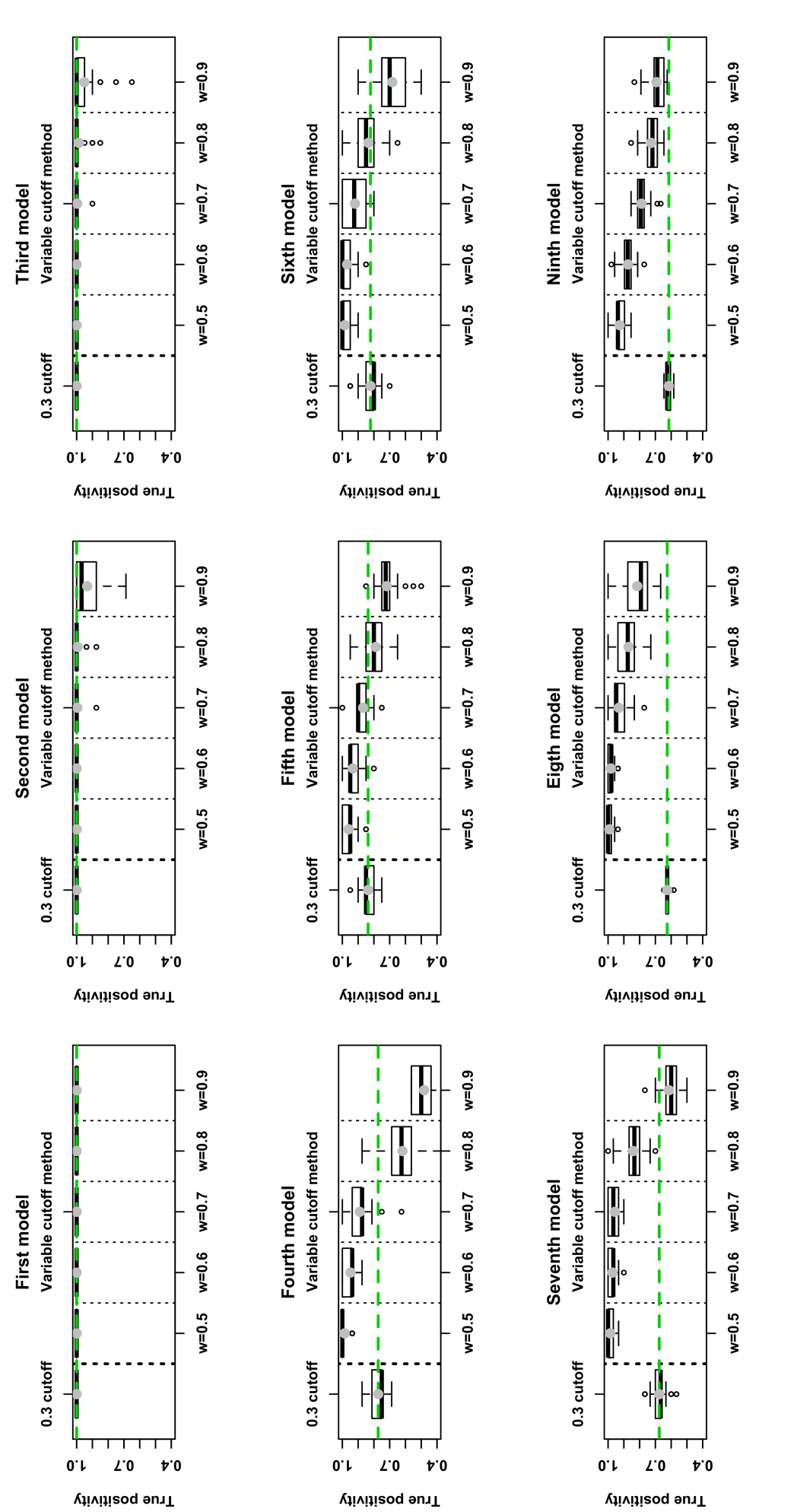


**Fig 3.** Boxplots per model of the estimated true positivity rates using the constant $0.3$ cutoff on factor loadings and the variable cutoff methods with $5$ values of $w$. Green dashed line represents the mean estimated true positivity rates using the constant $0.3$ cutoff. Grey dots represent mean estimated true positivity rates. Using $w=0.7$ the variable cutoff method outperforms the constant $0.3$ cutoff except in the cases of models two and three. However, these models are trivial and suit the $0.3$ cutoff perfectly. In more complex models especially models $7$ to $9$ the variable cutoff method substantially outperforms the constant $0.3$ cutoff.


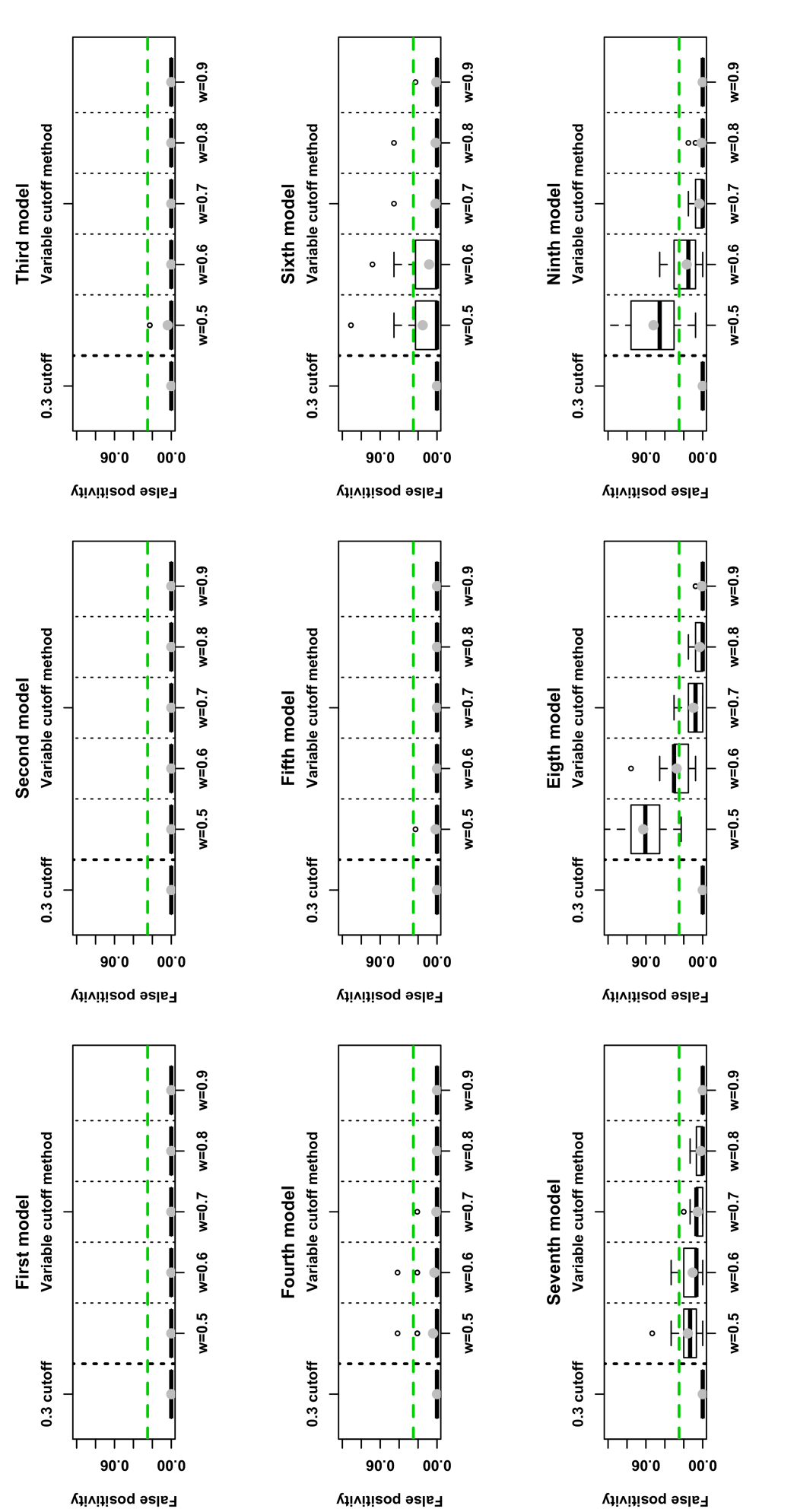
**Fig 4.**Boxplots per model of the estimated false positivity rates using the constant $0.3$ cutoff and the variable cutoff method. Green dashed line at 0 $0.025$. Grey dots represent mean estimated false positivity rates. Using $w=0.7$the variable cutoff method consistently produces mean estimated false positivity rates below $0.025$.


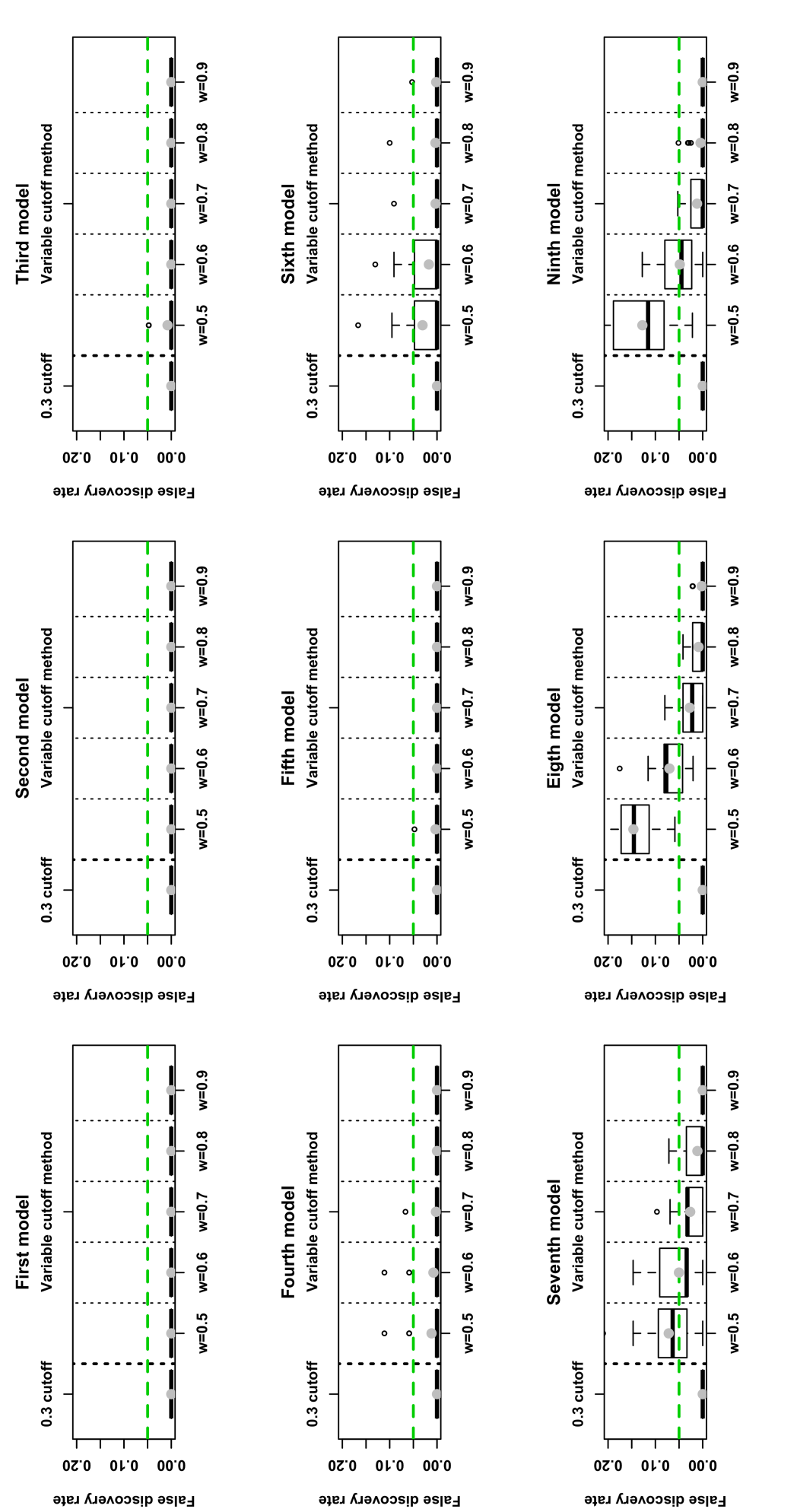
**Fig 5.** Boxplots per model of the estimated false discovery rates using the constant $0.3$ cutoff and the variable cutoff method. Green dashed line at $0.05$. Grey dots represent mean estimated false discovery rates. Using $w=0.7$ the variable cutoff method consistently produces mean estimated false discovery rates below $0.05$.

References

1. Spearman, C. General Intelligence, Objectively Determined and Mea- sured. Am J Psychol 1904;15:201–293.
2. Choi, J, Zou, H and Oehlert, G. A Penalized Maximum Likelihood Ap- proach to Sparse Factor Analysis. Stat Interface 2010:1–8.
3. Efron, B. Bootstrap methods: another look at the jackknife. Ann Stat 1979;7:1–26.
4. Buja, A and Eyuboglu, N. Remarks on Parallel Analysis. Multivariate Behav Res 1992;27:509–540.
5. Zientek, LR and Thompson, B. Applying the bootstrap to the multi- variate case: Bootstrap component/factor analysis. Behav Res Methods 2007;39:318–325.
6. Kaiser, HF. The Varimax Criterion For Analytic Rotation In Factor Analysis. Psychometrika 1958;23:187–200.
7. DiCiccio, TJ and Efron, B. Bootstrap Confidence Intervals. Stat Sci 1996;11:189–228.
