# Supplementary material for: Identifying radiation-induced survivorship syndromes affecting bowel health in a cohort of gynecological cancer survivors
Source: PLoS One. 2017 Feb 3;12(2):e0171461. doi: 10.1371/journal.pone.0171461 (PMC5291512; doi:10.1371/journal.pone.0171461)
Supplement: S1 Table — Possible radiation-induced pathophysiological processes in the suggested survivorship diseases. (DOCX) [file pone.0171461.s004.docx]

**Supplementary Table. Possible radiation-induced pathophysiological processes in the suggested survivorship diseases.**

| **Survivorship disease** | **Probable site for the disease** | **Possible pathophysiological processes caused by ionizing radiation** | **Remarks** |
| --- | --- | --- | --- |
| Anal-sphincter dysfunction | Anal sphincter | **I.** Continuous replacement of muscle fibers with connective tissue (fibrosis)[^1^](#_ENREF_1)  **II.** Immediate nerve-ending (receptor) damage[^2^](#_ENREF_2) | Any muscle receiving ionizing radiation may develop fibrosis.[^3^](#_ENREF_3) |
| Gut-wall dysfunction | Sigmoid, rectum | **I.** Tight junctions made dysfunctional[^4^](#_ENREF_4)  **II.** Acute eradication of stem cells and progenitor cells[^5^](#_ENREF_5)  **III.** As long as the gut wall is permeable translocation of fecal bacteria into the gut wall[^6^](#_ENREF_6)  **IV.** Inflammation in the gut wall initiated by pro-inflammatory bacteria[^7^](#_ENREF_7)  **V.** On-going inflammation hinders stem-cell migration and division, making the gut wall continuously permeable  **VI.** Normal gut-wall cells replaced by connective tissue (fibrosis)  **V.** Immediate nerve-ending (receptor) damage[^2^](#_ENREF_2) |  |
| Excessive  gas  discharge | Bowel, gut microbiota | **I.** Unknown process causing excessive production, or decreased metabolism and absorption of hydrogen, hydrogen sulfide or methane.  **II.** Hydrogen sulfide causing the bad odor of flatulence  **III.** Hydrogen sulfide causing pain by stimulating nociceptive pain receptors[^8^](#_ENREF_8) | Bacteria are not believed to be sensitive to ionizing radiation.  Normally gut microbiota, including *Clostridium, Methanobrevibacter* and *Desulfovibrio* produce hydrogen, methane and hydrogen sulfide, respectively.[^9^](#_ENREF_9)^,^[^10^](#_ENREF_10) The gases are metabolized by the microbiota, absorbed through the gut wall or released as flatulence.[^11^](#_ENREF_11)  Normally gases are part of the regulation of gut motility.[^11^](#_ENREF_11)  Gut gases have been proposed to influence gut epithelial secretion[^12^](#_ENREF_12) and to be able to modify inflammatory processes.[^13^](#_ENREF_13) |
| Excessive mucus discharge | Small bowel, gut microbiota | **I.** Unknown processes cause increased mucus production  **II.** Loss of mucin-degrading bacteria  **III.** Loss of mucus-degrading function in existing bacteria | Mucus protects the gut epithelium and facilitates the movement of feces.[^14^](#_ENREF_14) Normally mucus is excreted by Goblet cells in the stomach as well as the small and large bowel.[^15^](#_ENREF_15) The daily production in an adult has been estimated to be 10 liters.  *Akkermansia, Bacteroides, Bifidobacterium, Clostridium, Ruminococcus* and possibly other bacterial genera normally degrade mucus,[^16^](#_ENREF_16)^,^[^17^](#_ENREF_17) hindering fecal leakage of mucus. Dose-volume modelling shows a clear association between dose to the small bowel and anal leakage of mucus.[^18^](#_ENREF_18) |
| Excessive blood discharge | Rectum, anal canal | **I.** On-going inflammation  **II.** Ulcerations as end result of fibrosis  **III.** Telangiectasia as end result of fibrosis | If the main cause is ulcerations and telangiectasia in a fibrotic gut.[^19^](#_ENREF_19) |

**References**

1. Da Silva GM, Berho M, Wexner SD, et al. Histologic analysis of the irradiated anal sphincter. Dis Colon Rectum 2003;46:1492-7.

2. Loganathan A, Schloithe AC, Hutton J, et al. Pudendal nerve injury in men with fecal incontinence after radiotherapy for prostate cancer. Acta Oncol 2015;54:882-8.

3. Straub JM, New J, Hamilton CD, et al. Radiation-induced fibrosis: mechanisms and implications for therapy. J Cancer Res Clin Oncol 2015;141:1985-94.

4. Shukla PK, Gangwar R, Manda B, et al. Rapid Disruption of Intestinal Epithelial Tight Junction and Barrier Dysfunction by Ionizing Radiation in Mouse Colon in vivo: Protection by N-Acetyl L-Cysteine. Am J Physiol Gastrointest Liver Physiol 2016:ajpgi 00314 2015.

5. Potten CS. Extreme sensitivity of some intestinal crypt cells to X and gamma irradiation. Nature 1977;269:518-21.

6. Li XH, Ghosh SP, Ha CT, et al. Delta-tocotrienol protects mice from radiation-induced gastrointestinal injury. Radiat Res 2013;180:649-57.

7. Touchefeu Y, Montassier E, Nieman K, et al. Systematic review: the role of the gut microbiota in chemotherapy- or radiation-induced gastrointestinal mucositis - current evidence and potential clinical applications. Aliment Pharmacol Ther 2014;40:409-21.

8. Schemann M, Grundy D. Role of hydrogen sulfide in visceral nociception. Gut 2009;58:744-7.

9. Gibson GR, Cummings JH, Macfarlane GT. Competition for hydrogen between sulphate-reducing bacteria and methanogenic bacteria from the human large intestine. J Appl Bacteriol 1988;65:241-7.

10. McKay LF, Holbrook WP, Eastwood MA. Methane and hydrogen production by human intestinal anaerobic bacteria. Acta Pathol Microbiol Immunol Scand B 1982;90:257-60.

11. Pimentel M, Mathur R, Chang C. Gas and the microbiome. Curr Gastroenterol Rep 2013;15:356.

12. Schicho R, Krueger D, Zeller F, et al. Hydrogen sulfide is a novel prosecretory neuromodulator in the Guinea-pig and human colon. Gastroenterology 2006;131:1542-52.

13. Picton R, Eggo MC, Langman MJ, et al. Impaired detoxication of hydrogen sulfide in ulcerative colitis? Dig Dis Sci 2007;52:373-8.

14. Johansson ME, Sjovall H, Hansson GC. The gastrointestinal mucus system in health and disease. Nat Rev Gastroenterol Hepatol 2013;10:352-61.

15. Johansson ME, Hansson GC. The goblet cell: a key player in ischaemia-reperfusion injury. Gut 2013;62:188-9.

16. Derrien M, Vaughan EE, Plugge CM, et al. Akkermansia muciniphila gen. nov., sp. nov., a human intestinal mucin-degrading bacterium. Int J Syst Evol Microbiol 2004;54:1469-76.

17. Hoskins LC, Boulding ET. Mucin degradation in human colon ecosystems. Evidence for the existence and role of bacterial subpopulations producing glycosidases as extracellular enzymes. J Clin Invest 1981;67:163-72.

18. Thor M, Olsson CE, Oh JH, et al. Relationships between dose to the gastro-intestinal tract and patient-reported symptom domains after radiotherapy for localized prostate cancer. Acta Oncol 2015;54:1326-34.

19. Laterza L, Cecinato P, Guido A, et al. Management of radiation-induced rectal bleeding. Curr Gastroenterol Rep 2013;15:355.
